# Correction: A deep hybrid learning pipeline for accurate diagnosis of ovarian cancer based on nuclear morphology

**DOI:** 10.1371/journal.pone.0316114

**Published:** 2024-12-17

**Authors:** Duhita Sengupta, Sk Nishan Ali, Aditya Bhattacharya, Joy Mustafi, Asima Mukhopadhyay, Kaushik Sengupta

There is an error in affiliation 2 for the author Duhita Sengupta. The correct affiliation 2 is: Homi Bhabha National Institute, Mumbai, India

An additional affiliation is missing for the sixth author. Kaushik Sengupta is also affiliated with Homi Bhabha National Institute, Mumbai, India.
