# Supplementary material for: A deep hybrid learning pipeline for accurate diagnosis of ovarian cancer based on nuclear morphology
Source: PLoS One. 2022 Jan 7;17(1):e0261181. doi: 10.1371/journal.pone.0261181 (PMC8741040; doi:10.1371/journal.pone.0261181)
Supplement: S1 Fig — For the Incept Layer, it utilized the number of filters for the Conv2D sub-layer and another hyper-parameter for the number of filters for the Left and Right Conv2D sub-layer as input. In both the Conv2D sub-layers, after tuning, a learning rate of 0.1 and an activation function of Leaky ReLu were used to learn the non-linear relationships in the underlying high dimensional data. For the initial Conv2D layer, we have used a filter dimension of 5x5 and strided convolution with stride as 2. For Left Conv2D a filter size of 3x3 was used and for the Right Conv2D, a filter size of 5x5 was used. Finally, both the left and right conv2d sub-layers were concatenated and passed to the next layer. The Squeeze layer followed the same structure as Incept Layer. The learning rates and the activation function used in the sub-layers were the same, the only difference being with the filter dimensions. For the initial Conv2D sub-layer, the dimension was (1x1) with stride 1 while for Left Conv2D & Right Conv2D the filter dimensions were (1x1) and (3x3) respectively. Like the Incept layer, the Left and the Right Sub-layers were concatenated and passed to the next layers. After a series of Incept and Squeeze layers we used another Conv2D layer with 64 filters and each filter was of dimension 3x3, with an activation function of Leaky ReLu with a learning rate of 0.1. A combination of dropout and L2 regularization was used to reduce the chances of overfitting. Finally, after all the convolution layers which were used to extract the features, we flattened the output and passed the flattened output to classical Machine Learning algorithms like XGBoost and Random Forest for the final classification part. (DOCX) [file pone.0261181.s001.docx]

**A Deep Hybrid Learning pipeline for accurate diagnosis of Ovarian Cancer based on Nuclear Morphology**

**Duhita Sengupta^1¶^, Sk Nishan Ali^2¶^, Aditya Bhattacharya^2^, Joy Mustafi^2^, Asima Mukhopadhyay^3a#b#c#^ & Kaushik Sengupta^1^***

^1^Biophysics and Structural Genomics Division, Saha Institute of Nuclear Physics, 1/AF Bidhannagar, Kolkata, West Bengal 700064 India; HomiBhaba National Institute, Mumbai, India

^2^Artificial Intelligence and Machine Learning Division, MUST Research Trust, Hyderabad, 500046, Telangana, India

^3a#^Chittaranjan National Cancer Institute, Newtown, Kolkata, West Bengal 700156, India

^b#^Current Address: Northern Gynaecological Oncology Centre, Queen Elizabeth Hospital, Gateshead,NE9 6SX, United Kingdom

^c#^Formerly at Tata Medical Center, Kolkata, West Bengal 700156, India

^¶^Authors contributed equally

*To whom correspondence should be addressed: [kaushik.sengupta@saha.ac.in](mailto:kaushik.sengupta@saha.ac.in)

**Supplementary Figures:**


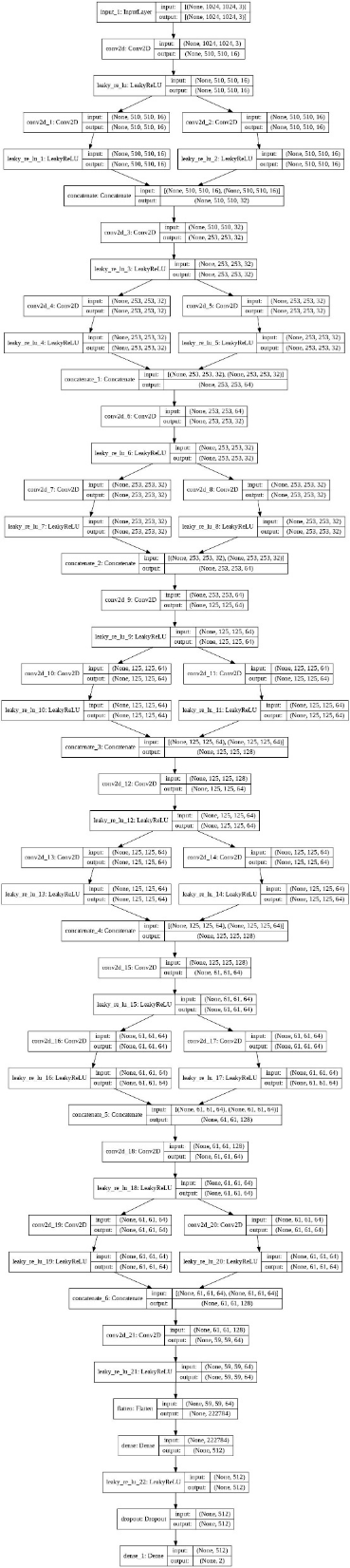


**S1 Fig: Network Diagram.** For the Incept Layer, it utilized the number of filters for the Conv2D sub-layer and another hyper-parameter for the number of filters for Left and Right Conv2D sub-layer as input. In both the Conv2D sub-layers, after tuning, a learning rate of 0.1 and an activation function of Leaky ReLu was used to learn the non-linear relationships in the underlying high dimensional data. For the initial Conv2D layer, we have used a filter dimension of 5x5 and strided convolution with stride as 2. For Left Conv2D a filter size of 3x3 was used and for the Right Conv2D a filter size of 5x5 was used. Finally, both the left and right conv2d sub layers were concatenated and passed to the next layer. The Squeeze layer followed the same structure as Incept Layer. The learning rates and the activation function used in the sub-layers were the same, the only differencebeing with the filter dimensions. For the initial Conv2D sub layer, the dimension was (1x1) with stride 1 while for Left Conv2D & Right Conv2D the filter dimensions were (1x1) and (3x3) respectively. Like the Incept layer, the Left and the Right Sub-layers were concatenated and passed to the next layers. After a series of Incept and Squeeze layers we used another Conv2D layer with 64 filters and each filter was of dimension 3x3, with an activation function of Leaky ReLu with learning rate 0.1. A combination of dropout and L2 regularization were used to reduce the chances of over fitting. Finally, after all the convolution layers which were used to extract the features, we flattened the output and passed the flattened output to classical Machine Learning algorithms like XGBoost and Random Forest for the final classification part.
