# Supplementary material for: A deep hybrid learning pipeline for accurate diagnosis of ovarian cancer based on nuclear morphology
Source: PLoS One. 2022 Jan 7;17(1):e0261181. doi: 10.1371/journal.pone.0261181 (PMC8741040; doi:10.1371/journal.pone.0261181)
Supplement: S2 Fig — The X-axis denotes the normalized number of nuclei with respect to the total number of nuclei calculated. Y-axis denotes the measure of the parameter. A. 1. Comparative distribution of the number of normal (Mean±Std error of mean:0.8796± 0.005994) (Std Dev:0.1285±0.00495) and ovarian cancer (Mean±Std error of mean:0.8452± 0.006767) (Std Dev:0.1347±0.004785) nuclei based on Circularity values acquired from lamin A stained tissues. A. 2. Comparative distribution of the number of normal (Mean±Std error of mean:0.9309± 0.003013) (Std Dev:0.8024±0.002131) and ovarian cancer (Mean±Std error of mean:0.8974± 0.005269) (Std Dev:0.1135±0.003726) nuclei based on Circularity values acquired from lamin B stained tissues. B. 1. Comparative distribution of the number of normal (Mean±Std error of mean:0.7067± 0.01169) (Std Dev:0.2144±0.008269) and ovarian cancer (Mean±Std error of mean:0.7885± 0.008241) (StdDev:0.164±0.005827) nuclei based on Eccentricity values acquired from lamin A stained tissues. B. 2. Comparative distribution of the number of normal (Mean±Std error of mean:0.6348± 0.008105) (Std Dev:0.2147±0.005731)and diseased (Mean±Std error of mean:0.7076± 0.008841) (Std Dev:0.19±0.006252)nuclei based on Eccentricity values acquired from lamin B stained tissues. C. 1. Comparative distribution of the number of normal (Mean±Std error of mean:5.204± 0.1648) (Std Dev:3.03±0.1165) and ovarian cancer (Mean±Std error of mean:8.942± 0.1729) (Std Dev:3.441±0.1223) nuclei based on Foci Distance values acquired from lamin A stained tissues. C. 2. Comparative distribution of the number of normal (Mean±Std error of mean:3.934± 0.07391) (Std Dev:1.968±0.05226) and diseased (Mean±Std error of mean:7.414± 0.1738) (Std Dev:3.744±0.1229)nuclei based on Foci Distance values acquired from lamin B stained tissues. D. 1. Comparative distribution of the number of normal (Mean±Std error of mean:12.17± 0.3004) (Std Dev:5.523±0.2124) and ovarian cancer (Mean±Std error of mean:20.01± 0.3152) (Std Dev [file pone.0261181.s002.docx]

**A Deep Hybrid Learning pipeline for accurate diagnosis of Ovarian Cancer based on Nuclear Morphology**

**Duhita Sengupta^1¶^, Sk Nishan Ali^2¶^, Aditya Bhattacharya^2^, Joy Mustafi^2^, Asima Mukhopadhyay^3a#b#c#^ & Kaushik Sengupta^1^***

^1^Biophysics and Structural Genomics Division, Saha Institute of Nuclear Physics, 1/AF Bidhannagar, Kolkata, West Bengal 700064 India; HomiBhaba National Institute, Mumbai, India

^2^Artificial Intelligence and Machine Learning Division, MUST Research Trust, Hyderabad, 500046, Telangana, India

^3a#^Chittaranjan National Cancer Institute, Newtown, Kolkata, West Bengal 700156, India

^b#^Current Address: Northern Gynaecological Oncology Centre, Queen Elizabeth Hospital, Gateshead,NE9 6SX, United Kingdom

^c#^Formerly at Tata Medical Center, Kolkata, West Bengal 700156, India

^¶^Authors contributed equally

*To whom correspondence should be addressed: [kaushik.sengupta@saha.ac.in](mailto:kaushik.sengupta@saha.ac.in)

**
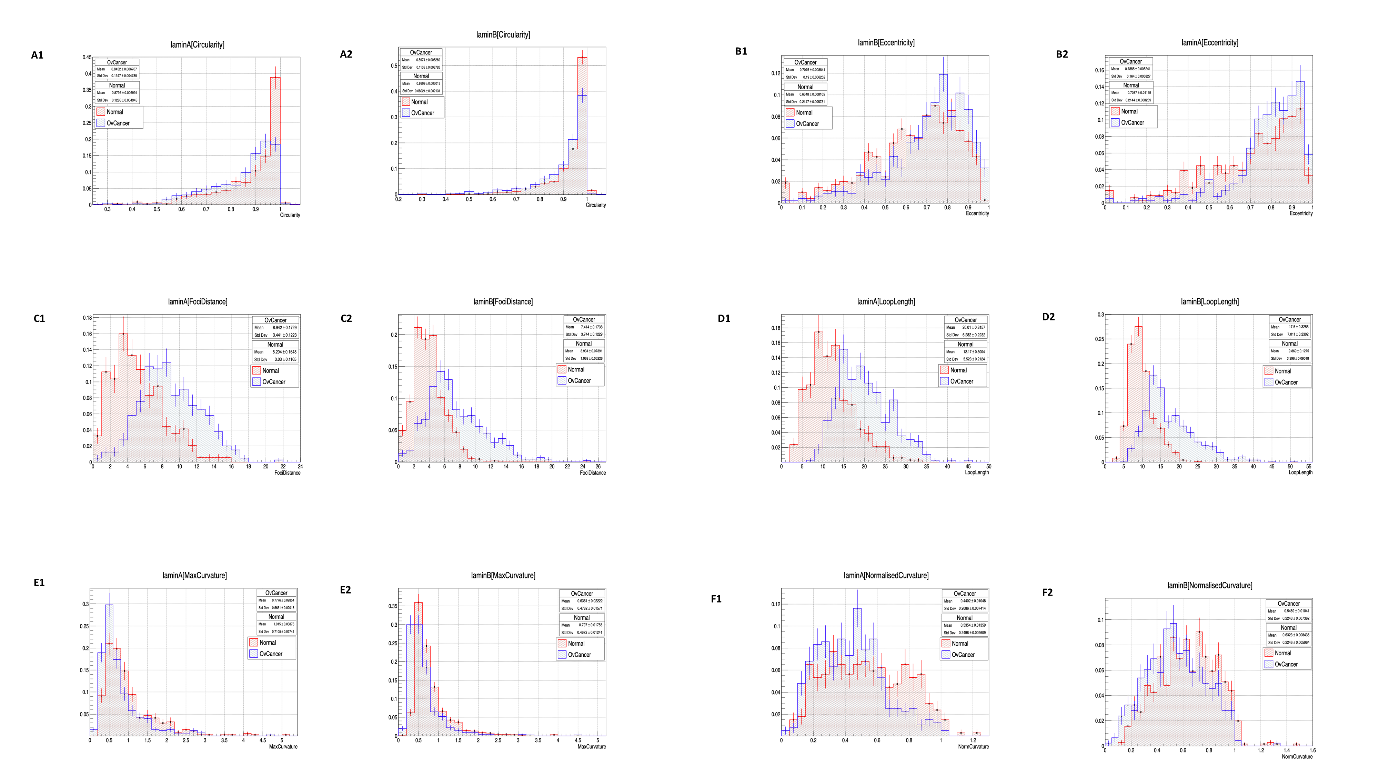
**

**S2 Fig: Histograms showing distributions of the normal and Ovarian Cancer nuclei based on different morphometric parameters obtained from lamin A and B stained tissue sample images before and after pre-processing.** X axis denotes the normalised number of nuclei with respect to the total number of nuclei calculated. Y axis denotes the measure of the parameter.

A. 1.Comparative distribution of the number of normal (Mean±Std error of mean:0.8796± 0.005994) (Std Dev:0.1285±0.00495) and ovarian cancer (Mean±Std error of mean:0.8452± 0.006767) (Std Dev:0.1347±0.004785) nuclei based on Circularity values acquired from lamin A stained tissues.

A. 2. Comparative distribution of the number of normal (Mean±Std error of mean:0.9309± 0.003013) (Std Dev:0.8024±0.002131) and ovarian cancer (Mean±Std error of mean:0.8974± 0.005269) (Std Dev:0.1135±0.003726) nuclei based on Circularity values acquired from laminB stained tissues.

B. 1.Comparative distribution of the number of normal (Mean±Std error of mean:0.7067± 0.01169) (Std Dev:0.2144±0.008269)  and ovarian cancer (Mean±Std error of mean:0.7885± 0.008241) (StdDev:0.164±0.005827) nuclei based on Eccentricity values acquired from lamin A stained tissues.

B. 2. Comparative distribution of the number of normal (Mean±Std error of mean:0.6348± 0.008105) (Std Dev:0.2147±0.005731)and diseased (Mean±Std error of mean:0.7076± 0.008841) (Std Dev:0.19±0.006252)nuclei based on Eccentricity values acquired from lamin B stained tissues.

C. 1.Comparative distribution of the number of normal (Mean±Std error of mean:5.204± 0.1648) (Std Dev:3.03±0.1165)  and ovarian cancer (Mean±Std error of mean:8.942± 0.1729) (Std Dev:3.441±0.1223) nuclei based on Foci Distance values acquired from lamin A stained tissues.

C. 2. Comparative distribution of the number of normal (Mean±Std error of mean:3.934± 0.07391) (Std Dev:1.968±0.05226) and diseased (Mean±Std error of mean:7.414± 0.1738) (Std Dev:3.744±0.1229)nuclei based on Foci Distance values acquired from lamin B stained tissues.

D. 1.Comparative distribution of the number of normal (Mean±Std error of mean:12.17± 0.3004) (Std Dev:5.523±0.2124)  and ovarian cancer (Mean±Std error of mean:20.01± 0.3152) (Std Dev:6.282±0.2232) nuclei based on Loop Length values acquired from lamin A stained tissues.

D. 2. Comparative distribution of the number of normal (Mean±Std error of mean:9.869± 0.1275) (Std Dev:3.396±0.09018)and diseased (Mean±Std error of mean:17.5± 0.3255) (Std Dev:7.011±0.2302)nuclei based on Loop Length values acquired from lamin B stained tissues.

E. 1.Comparative distribution of the number of normal (Mean±Std error of mean:1.1015± 0.03876) (Std Dev:0.7105±0.02741)  and ovarian cancer (Mean±Std error of mean:0.7776± 0.02854) (Std Dev:0.5651±0.02018) nuclei based on Maximum Curvature values acquired from lamin A stained tissues.

E. 2. Comparative distribution of the number of normal (Mean±Std error of mean:0.797± 0.01755) (Std Dev:0.4672±0.01241)and diseased (Mean±Std error of mean:0.6361± 0.0222) (Std Dev:0.4782±0.01571)nuclei based on Maximum Curvature values acquired from lamin B stained tissues.

F. 1.Comparative distribution of the number of normal (Mean±Std error of mean:0.5354± 0.01359) (Std Dev:0.2496±0.009609)  and ovarian cancer (Mean±Std error of mean:0.4402± 0.01048) (Std Dev:0.2086±0.007414nuclei based on Normalized Curvature values acquired from lamin A stained tissues.

F. 2. Comparative distribution of the number of normal (Mean±Std error of mean:0.6323± 0.008435) (Std Dev:0.2246±0.005964)and diseased (Mean±Std error of mean:0.5489± 0.01401) (Std Dev:0.2243±0.007362) nuclei based on Normalized Curvature values acquired from lamin B stained tissues.
