# Supplementary material for: A deep hybrid learning pipeline for accurate diagnosis of ovarian cancer based on nuclear morphology
Source: PLoS One. 2022 Jan 7;17(1):e0261181. doi: 10.1371/journal.pone.0261181 (PMC8741040; doi:10.1371/journal.pone.0261181)
Supplement: S4 Fig — PCA was used further to minimize correlation. (DOCX) [file pone.0261181.s004.docx]

**A Deep Hybrid Learning pipeline for accurate diagnosis of Ovarian Cancer based on Nuclear Morphology**

**Duhita Sengupta^1¶^, Sk Nishan Ali^2¶^, Aditya Bhattacharya^2^, Joy Mustafi^2^, Asima Mukhopadhyay^3a#b#c#^ & Kaushik Sengupta^1^***

^1^Biophysics and Structural Genomics Division, Saha Institute of Nuclear Physics, 1/AF Bidhannagar, Kolkata, West Bengal 700064 India; HomiBhaba National Institute, Mumbai, India

^2^Artificial Intelligence and Machine Learning Division, MUST Research Trust, Hyderabad, 500046, Telangana, India

^3a#^Chittaranjan National Cancer Institute, Newtown, Kolkata, West Bengal 700156, India

^b#^Current Address: Northern Gynaecological Oncology Centre, Queen Elizabeth Hospital, Gateshead,NE9 6SX, United Kingdom

^c#^Formerly at Tata Medical Center, Kolkata, West Bengal 700156, India

^¶^Authors contributed equally

*To whom correspondence should be addressed: [kaushik.sengupta@saha.ac.in](mailto:kaushik.sengupta@saha.ac.in)

**
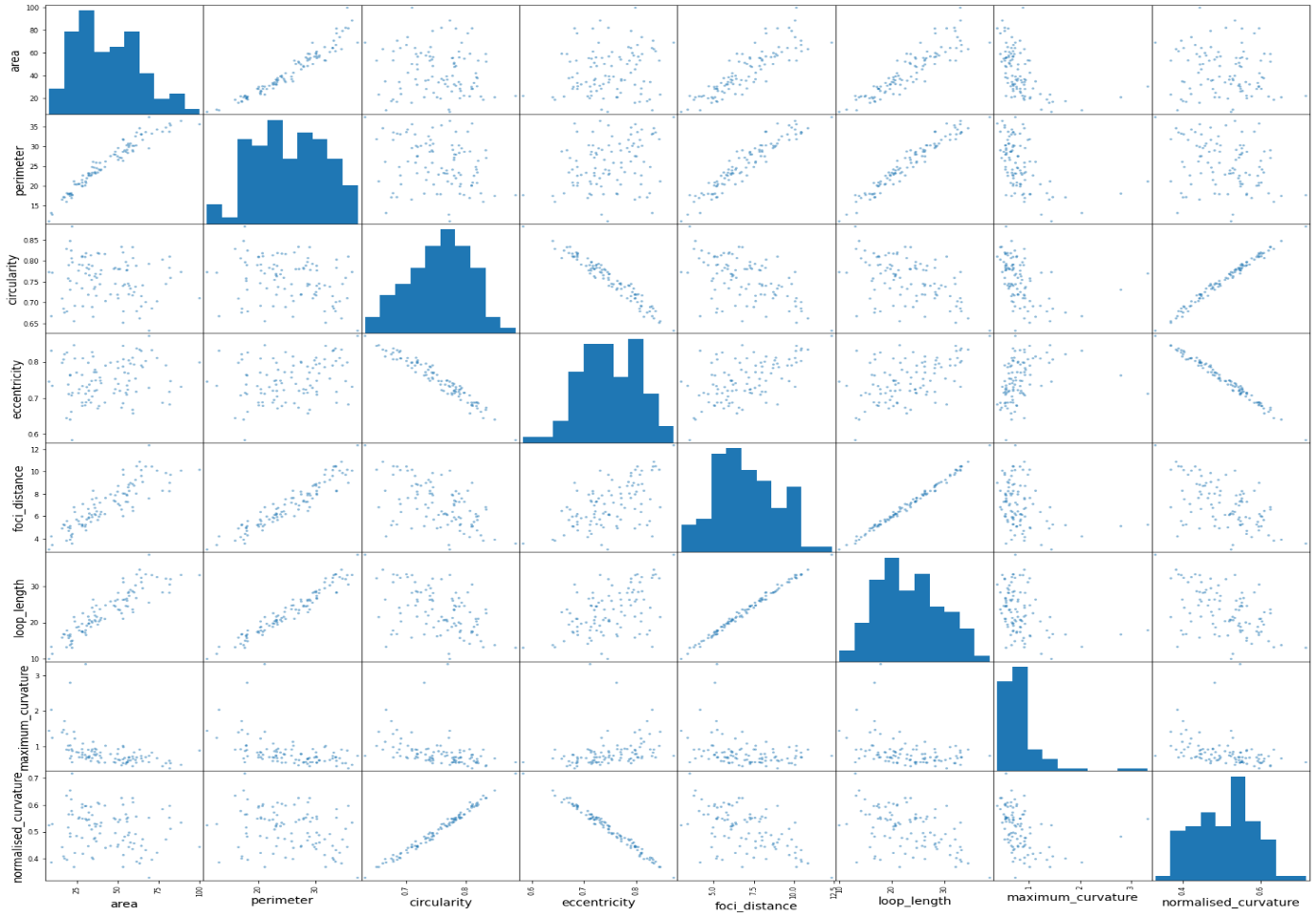
**

**S4 Fig:****Correlation Matrix:** Correlation Matrix to analyze how the output variable is correlated with the other parameters. PCA was used further to minimize correlation
