# Supplementary material for: A deep hybrid learning pipeline for accurate diagnosis of ovarian cancer based on nuclear morphology
Source: PLoS One. 2022 Jan 7;17(1):e0261181. doi: 10.1371/journal.pone.0261181 (PMC8741040; doi:10.1371/journal.pone.0261181)
Supplement: S2 Table — (DOCX) [file pone.0261181.s006.docx]

**A Deep Hybrid Learning pipeline for accurate diagnosis of Ovarian Cancer based on Nuclear Morphology**

**Duhita Sengupta^1¶^, Sk Nishan Ali^2¶^, Aditya Bhattacharya^2^, Joy Mustafi^2^, Asima Mukhopadhyay^3a#b#c#^ & Kaushik Sengupta^1^***

^1^Biophysics and Structural Genomics Division, Saha Institute of Nuclear Physics, 1/AF Bidhannagar, Kolkata, West Bengal 700064 India; HomiBhaba National Institute, Mumbai, India

^2^Artificial Intelligence and Machine Learning Division, MUST Research Trust, Hyderabad, 500046, Telangana, India

^3a#^Chittaranjan National Cancer Institute, Newtown, Kolkata, West Bengal 700156, India

^b#^Current Address: Northern Gynaecological Oncology Centre, Queen Elizabeth Hospital, Gateshead,NE9 6SX, United Kingdom

^c#^Formerly at Tata Medical Center, Kolkata, West Bengal 700156, India

^¶^Authors contributed equally

*To whom correspondence should be addressed: [kaushik.sengupta@saha.ac.in](mailto:kaushik.sengupta@saha.ac.in)

**S6 Table: Model layers and dimensions**

Layer (type) Output Shape Param # Connected to

=================================================================================================

input_1 (InputLayer) [(None, 1024, 1024, 0

| conv2d (Conv2D) | (None, | 510, | 510, | 16) | 1216 | input_1[0][0] |
| --- | --- | --- | --- | --- | --- | --- |
| leaky_re_lu (LeakyReLU) | (None, | 510, | 510, | 16) | 0 | conv2d[0][0] |
| conv2d_1 (Conv2D) | (None, | 510, | 510, | 16) | 2320 | leaky_re_lu[0][0] |
| conv2d_2 (Conv2D) | (None, | 510, | 510, | 16) | 6416 | leaky_re_lu[0][0] |
| leaky_re_lu_1 (LeakyReLU) | (None, | 510, | 510, | 16) | 0 | conv2d_1[0][0] |
| leaky_re_lu_2 (LeakyReLU) | (None, | 510, | 510, | 16) | 0 | conv2d_2[0][0] |
| concatenate (Concatenate) | (None, | 510, | 510, | 32) | 0 | leaky_re_lu_1[0][0]  leaky_re_lu_2[0][0] |
| conv2d_3 (Conv2D) | (None, | 253, | 253, | 32) | 25632 | concatenate[0][0] |
| leaky_re_lu_3 (LeakyReLU) | (None, | 253, | 253, | 32) | 0 | conv2d_3[0][0] |
| conv2d_4 (Conv2D) | (None, | 253, | 253, | 32) | 9248 | leaky_re_lu_3[0][0] |
| conv2d_5 (Conv2D) | (None, | 253, | 253, | 32) | 25632 | leaky_re_lu_3[0][0] |
| leaky_re_lu_4 (LeakyReLU) | (None, | 253, | 253, | 32) | 0 | conv2d_4[0][0] |
| leaky_re_lu_5 (LeakyReLU) | (None, | 253, | 253, | 32) | 0 | conv2d_5[0][0] |
| concatenate_1 (Concatenate) | (None, | 253, | 253, | 64) | 0 | leaky_re_lu_4[0][0]  leaky_re_lu_5[0][0] |
| conv2d_6 (Conv2D) | (None, | 253, | 253, | 32) | 2080 | concatenate_1[0][0] |
| leaky_re_lu_6 (LeakyReLU) | (None, | 253, | 253, | 32) | 0 | conv2d_6[0][0] |
| conv2d_7 (Conv2D) | (None, | 253, | 253, | 32) | 1056 | leaky_re_lu_6[0][0] |
| conv2d_8 (Conv2D) | (None, | 253, | 253, | 32) | 9248 | leaky_re_lu_6[0][0] |
| leaky_re_lu_7 (LeakyReLU) | (None, | 253, | 253, | 32) | 0 | conv2d_7[0][0] |
| leaky_re_lu_8 (LeakyReLU) | (None, | 253, | 253, | 32) | 0 | conv2d_8[0][0] |

| concatenate_2 (Concatenate) | (None, | 253, | 253, 64) | 0 | leaky_re_lu_7[0][0]  leaky_re_lu_8[0][0] |
| --- | --- | --- | --- | --- | --- |
| conv2d_9 (Conv2D) | (None, | 125, | 125, 64) | 102464 | concatenate_2[0][0] |
| leaky_re_lu_9 (LeakyReLU) | (None, | 125, | 125, 64) | 0 | conv2d_9[0][0] |
| conv2d_10 (Conv2D) | (None, | 125, | 125, 64) | 36928 | leaky_re_lu_9[0][0] |
| conv2d_11 (Conv2D) | (None, | 125, | 125, 64) | 102464 | leaky_re_lu_9[0][0] |
| leaky_re_lu_10 (LeakyReLU) | (None, | 125, | 125, 64) | 0 | conv2d_10[0][0] |
| leaky_re_lu_11 (LeakyReLU) | (None, | 125, | 125, 64) | 0 | conv2d_11[0][0] |
| concatenate_3 (Concatenate) | (None, | 125, | 125, 128 | 0 | leaky_re_lu_10[0][0]  leaky_re_lu_11[0][0] |
| conv2d_12 (Conv2D) | (None, | 125, | 125, 64) | 8256 | concatenate_3[0][0] |
| leaky_re_lu_12 (LeakyReLU) | (None, | 125, | 125, 64) | 0 | conv2d_12[0][0] |
| conv2d_13 (Conv2D) | (None, | 125, | 125, 64) | 4160 | leaky_re_lu_12[0][0] |
| conv2d_14 (Conv2D) | (None, | 125, | 125, 64) | 36928 | leaky_re_lu_12[0][0] |
| leaky_re_lu_13 (LeakyReLU) | (None, | 125, | 125, 64) | 0 | conv2d_13[0][0] |
| leaky_re_lu_14 (LeakyReLU) | (None, | 125, | 125, 64) | 0 | conv2d_14[0][0] |
| concatenate_4 (Concatenate) | (None, | 125, | 125, 128 | 0 | leaky_re_lu_13[0][0]  leaky_re_lu_14[0][0] |
| conv2d_15 (Conv2D) | (None, | 61, | 61, 64) | 204864 | concatenate_4[0][0] |
| leaky_re_lu_15 (LeakyReLU) | (None, | 61, | 61, 64) | 0 | conv2d_15[0][0] |
| conv2d_16 (Conv2D) | (None, | 61, | 61, 64) | 36928 | leaky_re_lu_15[0][0] |
| conv2d_17 (Conv2D) | (None, | 61, | 61, 64) | 102464 | leaky_re_lu_15[0][0] |
| leaky_re_lu_16 (LeakyReLU) | (None, | 61, | 61, 64) | 0 | conv2d_16[0][0] |
| leaky_re_lu_17 (LeakyReLU) | (None, | 61, | 61, 64) | 0 | conv2d_17[0][0] |
| concatenate_5 (Concatenate) | (None, | 61, | 61, 128) | 0 | leaky_re_lu_16[0][0]  leaky_re_lu_17[0][0] |
